# Artificial neural networks improve LDCT lung cancer screening: a comparative validation study

**DOI:** 10.1186/s12885-020-07465-1

**Published:** 2020-10-22

**Authors:** Yin-Chen Hsu, Yuan-Hsiung Tsai, Hsu-Huei Weng, Li-Sheng Hsu, Ying-Huang Tsai, Yu-Ching Lin, Ming-Szu Hung, Yu-Hung Fang, Chien-Wei Chen

**Affiliations:** 1 Department of Diagnostic Radiology, Chang Gung Memorial Hospital Chiayi Branch, Chiayi, Taiwan; 2 Department of Medicine, Chang Gung University College of Medicine, Taoyuan, Taiwan; 3 Department of Biomedical Engineering, National Cheng Kung University, Tainan, Taiwan; 4 Department of Pulmonary and Critical Care Medicine, Chang Gung Memorial Hospital, Linkou, Taiwan; 5 Department of Respiratory Therapy, Chang Gung University, Taoyuan, Taiwan; 6 Department of Pulmonary and Critical Care Medicine, Chang Gung Memorial Hospital Chiayi Branch, Chiayi, Taiwan; 7 Department of Respiratory Care, Chang Gung University of Science and Technology Chiayi Campus, Chiayi, Taiwan; 8 Department of Respiratory Care, Chang Gung Memorial Hospital Chiayi Branch, Chiayi, Taiwan; 9 Institute of Medicine, Chung Shan Medical University, Taichung, Taiwan; 10 Department of Medical Imaging and Radiology, Shu-Zen Junior College of Medicine and Management, Kaohsiung, Taiwan

**Keywords:** Early detection of cancer, Receiver operating characteristic (ROC) curves, Sensitivity and specificity, Machine learning, Data visualization

## Abstract

**Background:**

This study proposes a prediction model for the automatic assessment of lung cancer risk based on an artificial neural network (ANN) with a data-driven approach to the low-dose computed tomography (LDCT) standardized structure report.

**Methods:**

This comparative validation study analysed a prospective cohort from Chiayi Chang Gung Memorial Hospital, Taiwan. In total, 836 asymptomatic patients who had undergone LDCT scans between February 2017 and August 2018 were included, comprising 27 lung cancer cases and 809 controls. A derivation cohort of 602 participants (19 lung cancer cases and 583 controls) was collected to construct the ANN prediction model. A comparative validation of the ANN and Lung-RADS was conducted with a prospective cohort of 234 participants (8 lung cancer cases and 226 controls). The areas under the curves (AUCs) of the receiver operating characteristic (ROC) curves were used to compare the prediction models.

**Results:**

At the cut-off of category 3, the Lung-RADS had a sensitivity of 12.5%, specificity of 96.0%, positive predictive value of 10.0%, and negative predictive value of 96.9%. At its optimal cut-off value, the ANN had a sensitivity of 75.0%, specificity of 85.0%, positive predictive value of 15.0%, and negative predictive value of 99.0%. The area under the ROC curve was 0.764 for the Lung-RADS and 0.873 for the ANN (*P* = 0.01). The two most important predictors used by the ANN for predicting lung cancer were the documented sizes of partially solid nodules and ground-glass nodules.

**Conclusions:**

Compared to the Lung-RADS, the ANN provided better sensitivity for the detection of lung cancer in an Asian population. In addition, the ANN provided a more refined discriminative ability than the Lung-RADS for lung cancer risk stratification with population-specific demographic characteristics. When lung nodules are detected and documented in a standardized structured report, ANNs may better provide important insights for lung cancer prediction than conventional rule-based criteria.

## Background

Lung cancer is the leading cause of cancer mortality worldwide [1]. The National Lung Screening Trial (NLST) showed that low-dose computed tomography (LDCT) screening could reduce lung cancer mortality by 20% compared to chest X-ray (CXR) [2]. With the increasing use of LDCT for lung cancer screening, the American College of Radiology (ACR) introduced the Lung Imaging Screening Reporting and Data System (Lung-RADS), which assigns groups for screening populations [3]. Aimed at high-risk smokers in the USA, the validity of the Lung-RADS remains unclear in areas with a high prevalence of non-smoking-related lung cancer, such as China, Taiwan, and Japan [4]. In Taiwan, more than 95% of lung cancer patients are non-smokers, most of whom have adenocarcinoma [5, 6]. Given the wide range of lung cancer demographics in Asia, the implementation of the Lung-RADS is not yet universal [7]. To address ambiguity, medical institutions have developed various structured reporting systems [8]. However, there is no current evidence showing explicit superiority for any reporting system in assessing lung cancer risks.

The artificial neural network (ANN) is a field of artificial intelligence technology characterized by simulating biological neural systems based on mathematical theories [9]. ANNs modify their behaviour by adjusting the weights between hidden units until the output correctly converges to the ground truth, and they are particularly adept at classification problems with different input data [10]. With the ability to analyse complex nonlinear relationships between predictors and diseases, well-trained ANNs make predictions with greater accuracy than conventional rule-based criteria [11].

This study aims to propose a reporting system based on an ANN with a data-driven approach to the LDCT standardized structured report. We further explore determinants for predicting lung cancer in this study population.

## Methods

### Study design and participants

**Fig. 1:**
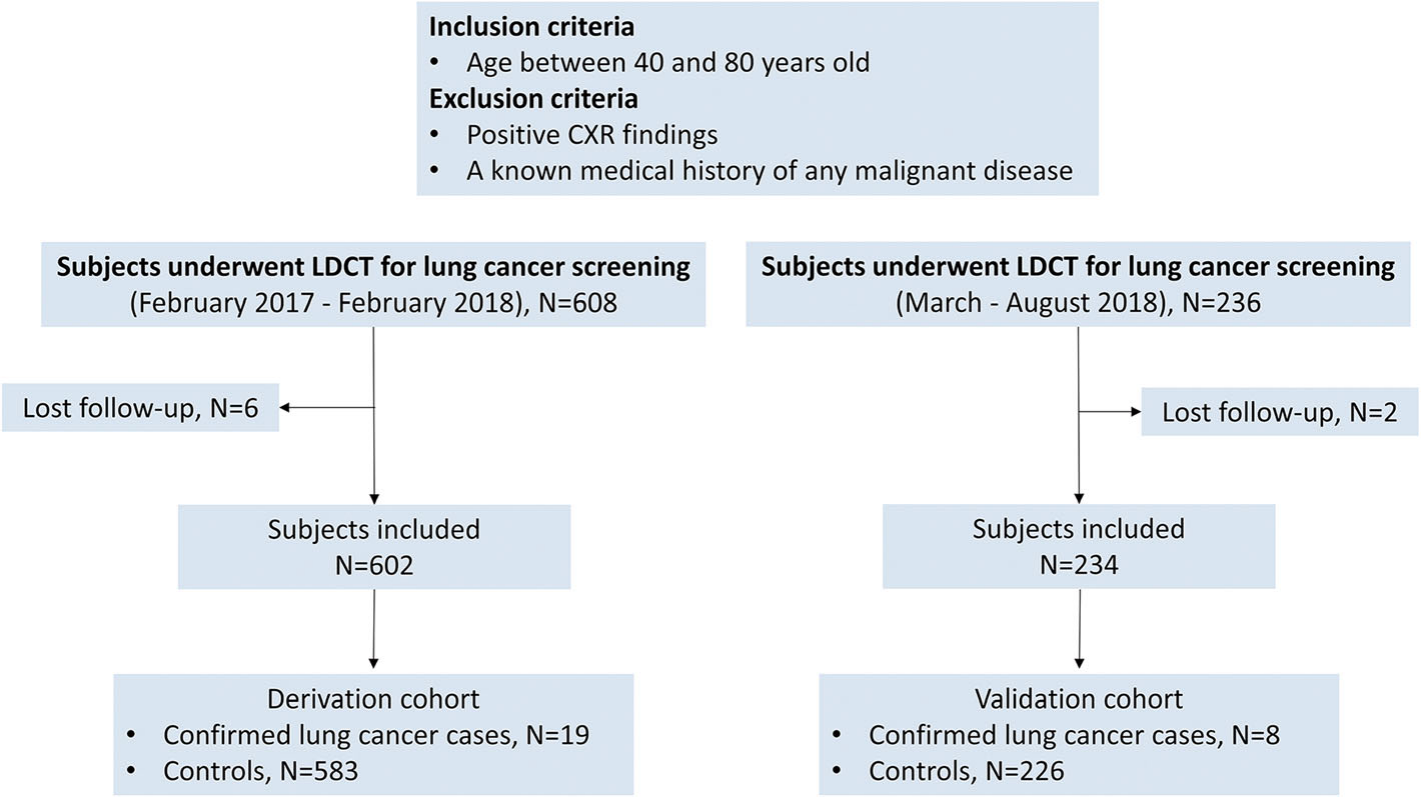
Flow diagram

The Institutional Review Board of Chang Gung Medical Foundation approved this case-control study. From February 2017 through August 2018, a total of 836 consecutive asymptomatic participants who underwent both CXR and LDCT at Chiayi Chang Gung Memorial Hospital, Taiwan, for lung cancer screening were prospectively enrolled. The inclusion criteria were age between 40 and 80 years old and willingness to participate in follow-up imaging or diagnostic workup. Subjects were excluded if a pulmonary nodule was detected on CXR, or if they had a known medical history of any malignant disease. Serial imaging reports, basic patient information, and demographic data were obtained. Each participant had at least 1 year of follow-up after the LDCT baseline scan. The diagnosis of lung cancer was confirmed based on surgical resection or lung biopsy and was recorded in a hospital-based cancer registry. Patients who had confirmed lung cancer prior to the index date of July 30, 2019 were classified as lung cancer patients (category 1); all other patients were classified as controls (category 0). Figure 1 shows the flowchart of the study.

### LDCT image acquisition and interpretation

All LDCT scans were performed with a 64-slice multidetector computed tomography (CT) (Somatom Sensation 64; Siemens Healthcare, Erlangen, Germany) in a low-dose setting without contrast enhancement (volumetric CT dose index ≤2.0 mGy for a standard patient). The scan parameters were 120 kVp, 25 effective mAs, soft-tissue kernel (B30f), and 3 mm slice thickness. All equipment specifications and acquisition parameters followed the recommendations of the ACR Society of Thoracic Radiology Practice Parameters for the Performance and Reporting of Lung Cancer Screening Thoracic CT [12]. Each LDCT baseline scan was reported by one thoracic radiologist with 7 years of experience. The standardized structured reports described the size, shape, location, and texture of the lung nodules, as well as other incidental findings. The density of each lung nodule was reported according to the definition from the Fleischner Society guidelines [13, 14]. The size of each lung nodule was measured on lung windows and recorded as recommended by the Lung-RADS.

### Development of the ANN

Each baseline LDCT report consists of a description of the intra- and extra-pulmonary findings, and a Lung-RADS risk category. The reports were designed to aid lung cancer screening. Using data scraping techniques, 22 input features were automatically extracted from the descriptive parts of the baseline LDCT reports and used to develop the ANN. Four of the inputs constituted clinical information or LDCT parameters. Another seven inputs pertained to nodule patterns and sizes based on the Lung-RADS standardized lexicon. The remaining inputs were extra-pulmonary interpretations, which consisted of 11 descriptive features. These inputs were in binary form (0 or 1). The Lung-RADS classification was not included among the input features. Table 1 lists all 22 input features, and shows the distribution of the baseline Lung-RADS categories in the derivation and validation cohorts.

**Table 1:** Clinical descriptors of the derivation and validation cohorts at the baseline

	Derivation cohort (***N*** = 602)		Validation cohort (***N*** = 234)		***P*** ^b^
	Cancer (***N*** = 19)	Control ^**c**^ (***N*** = 583)	Cancer (***N*** = 8)	Control ^**c**^ (***N*** = 226)	
Sex ^a^					
Male	7 (36.84%)	236 (40.48%)	2 (25.00%)	111 (49.12%)	0.038
Female	12 (63.16%)	347 (59.52%)	6 (75.00%)	115 (50.88%)	
Age (y) ^a^	64.89 ± 7.53	61.87 ± 6.42	57.63 ± 8.73	61.05 ± 7.88	0.053
LDCT parameters					
Dose (mSv) ^a^	1.95 ± 0.64	1.46 ± 0.24	1.78 ± 0.45	1.49 ± 0.26	0.161
DLP (mGy.cm) ^a^	75.53 ± 32.54	49.17 ± 11.08	64.50 ± 24.43	50.77 ± 10.72	0.206
Pattern of nodules					
Nodules of interest ^a^	2.42 (1–7)	1.11 (0–32)	1.88 (1–7)	1.29 (0–8)	0.330
Number of involved lobes ^a^	1.68 (1–3)	0.75 (0–5)	1.38 (1–4)	0.99 (0–5)	0.007
Size of nodules (mm)					
Solid nodule ^a^	10.01 (0–136.00)	1.49 (0–19.80)	0.63 (0–5.00)	1.80 (0–36.75)	1.000
PS nodule ^a^	3.89 (0–20.40)	0.38 (0–11.95)	1.77 (0–4.90)	0.54 (0–7.30)	0.498
GGN ^a^	8.87 (0–31.00)	0.58 (0–23.30)	4.56 (0–10.30)	0.24 (0–9.05)	0.038
Calcified nodule ^a^	0.00 (0)	0.39 (0–19.25)	0.86 (0–6.90)	0.55 (0–7.05)	0.100
Fat-containing nodule ^a^	0.00 (0)	0.05 (0–28.15)	0.00 (0)	0.00 (0)	0.506
Intra-pulmonary findings					
Linear atelectasis ^a^	10 (52.63%)	431 (73.93%)	2 (25.00%)	108 (47.79%)	< 0.001
Plate-like atelectasis ^a^	5 (26.32%)	73 (12.52%)	0 (0.00%)	19 (8.41%)	0.050
Plate-like GGN ^a^	2 (10.53%)	143 (24.53%)	1 (12.50%)	39 (17.26%)	0.029
Bronchiectasis ^a^	0 (0.00%)	39 (6.69%)	1 (12.50%)	8 (3.54%)	0.143
Emphysema ^a^	1 (5.26%)	51 (8.75%)	2 (25.00%)	28 (12.39%)	0.068
Fibrotic change ^a^	2 (10.53%)	154 (26.42%)	0 (0.00%)	42 (18.58%)	0.015
Extra-pulmonary findings					
Mediastinal tumour ^a^	4 (21.05%)	30 (5.15%)	1 (12.50%)	8 (3.54%)	0.290
Thyroid nodule ^a^	1 (5.26%)	19 (3.26%)	0 (0.00%)	2 (0.88%)	0.045
Adrenal nodule ^a^	1 (5.26%)	5 (0.86%)	0 (0.00%)	0 (0.00%)	0.125
Hepatic nodule ^a^	1 (5.26%)	67 (11.49%)	0 (0.00%)	20 (8.85%)	0.245
Renal nodule ^a^	0 (0.00%)	16 (2.74%)	0 (0.00%)	10 (4.42%)	0.229
Lung-RADS					
Category 1	0 (0.00%)	323 (55.40%)	0 (0.00%)	115 (50.89%)	0.240
Category 2	6 (31.58%)	222 (38.08%)	7 (87.50%)	102 (45.13%)	0.021
Category 3	5 (26.32%)	31 (5.32%)	1 (12.50%)	6 (2.65%)	0.080
Category 4	8 (42.10%)	7 (1.20%)	0 (0.00%)	3 (1.33%)	0.279

^a^ The 22 input features for developing the ANN

^b^ Comparison of the derivation cohort and validation cohort, *P*-values less than 0.05 indicated statistical significance

^c^ Participant who did not have confirmed lung cancer prior to the index date were labelled as control

*BMI* body mass index; *DLP* dose length product; *GGN* ground-glass nodule; *PS* nodule, part-solid nodule

The values are given as the mean ± SD, range or n (%)

**Fig. 2:**
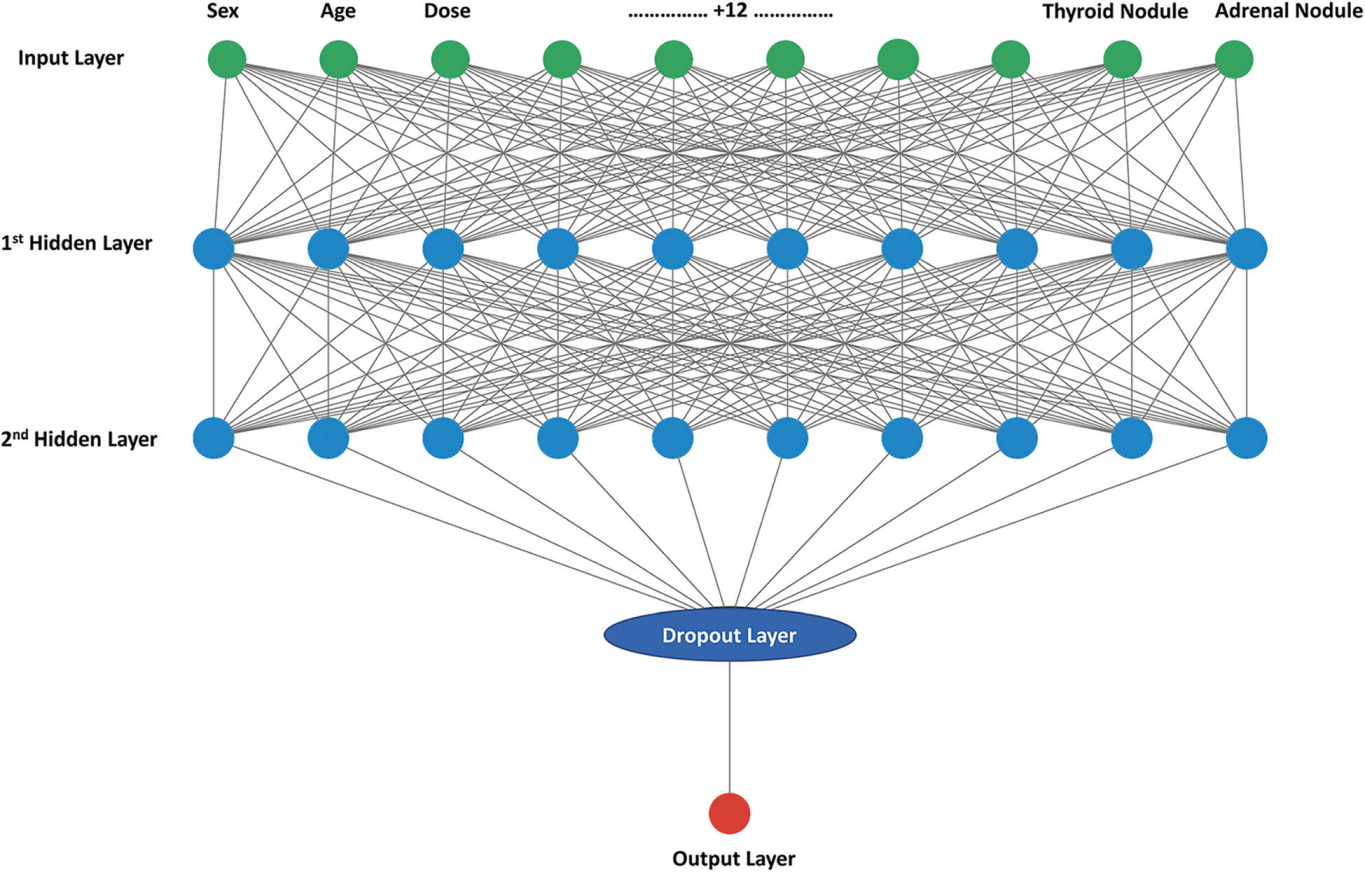
Structure of an ANN

Feed-forward neural networks based on the back-propagation algorithm were constructed using Keras version 2.2.4 [15], a high-level neural network application programming interface that can simplify the ANN construction process. The inputs for the ANN were normalized such that they fell between 0 and 1. The ANN consisted of the first two hidden layers, followed by a dropout layer to prevent over-fitting and a dense layer as the output layer [16]. There were 10 hidden units in each of the first two hidden layers and a rectified linear unit was used as the activation function. We also tested networks including different numbers of hidden units in each layer; none of these proved superior to the 10-unit network. Figure 2 shows the structure of the ANN. An adaptive learning rate optimizer based on the adaptive moment estimation method was used to facilitate convergence [17]. The network weights were randomly initialized between − 1 and 1. The learning rate was 0.001 and the dropout rate of the dropout layer was set to 0.1. The output layer eventually generated a number between 0 and 1 using the sigmoidal activation function. The predictive performance of the models was monitored during training to optimize the hyperparameters.

The dataset used in this study is unbalanced, but ANNs are sensitive to such datasets. Due to the iterative nature of the training, ANNs are prone to converge to the majority class. Thus, to achieve a cost-sensitive neural network, we used the class weighting approach; this assigns error weights to samples based on their class [18]. A 2:1 class weight ratio between lung cancer cases (category 1) and controls (category 0) was used in the ANN. We also explored networks with other class weight ratios (5:1, 10:1, 20:1, 25:1, 29:1 and 35:1). However, in terms of sensitivity, specificity and AUC, none of which performed significantly better than the setting with a 2:1 class weight ratio.

### Validation a*n*d risk group identification

In the training process, the ANN was internally validated via “three-fold cross-validation” [19]. The dataset was divided into three equal parts. At each cycle, one of the three parts was selected as the test set and removed from the dataset, while the remaining cases were used as the training set of the ANN. This process was repeated until the entire dataset had been used once as the test set. Finally, the ANN was validated with the prospective validation cohort.

To investigate the determining factors for predicting lung cancer, we applied a permutation feature importance method proposed by Leo Breiman [20]. The permutation feature importance for each feature used in the ANN was evaluated with the validation cohort, and the performance metric was AUC. At each iteration, one of the features was randomly shuffled, and the permutation feature importance score was calculated to show how much the performance metric decreased. Therefore, a high score revealed a feature with a great contribution to the discriminative ability of the model.

### Statistical analyses

Statistical analyses were performed using MedCalc 18.9.1 (MedCalc Software, Ostend, Belgium). Observed distributions were tested against the hypothesized normal distribution (Kolmogorov–Smirnov test). Data are reported as the mean ± standard deviation or number (%) unless otherwise indicated. To determine and compare the performance of the Lung-RADS and ANN, the sensitivity and specificity of the lung cancer classification at different thresholds were analysed based on the results of area under the receiver operating characteristic (ROC) curve analyses. The optimal diagnostic thresholds of the ROC curves were determined using maximized Youden’s [21] index. ROC curves were compared using the method described by DeLong et al. [22]. The sensitivity, specificity, positive predictive value (PPV), negative predictive value (NPV), positive likelihood ratio (LR+), and negative likelihood ratio (LR–) of each model for lung cancer diagnosis were calculated [23]. In all analyses, *P* < 0.05 was considered to indicate statistical significance.

## Results

### Demographic and clinical characteristics

The study cohort included a total of 836 consecutive asymptomatic participants who had undergone LDCT for lung cancer screening (27 lung cancer cases and 809 controls) at our institution. Between February 2017 and February 2018, 602 participants were included in the derivation cohort. Among the participants in the derivation cohort, 29 subjects underwent surgical resection or biopsy for tissue sampling. Nineteen of those subjects were diagnosed with lung cancer (adenocarcinoma in situ, *n* = 3; minimally invasive adenocarcinoma, *n* = 1; invasive adenocarcinoma, *n* = 14; small cell carcinoma, n = 1), and the remaining ten had benign lesions (pneumonia, *n* = 5; pulmonary fibrosis, *n* = 4; and pulmonary hamartoma, *n* = 1). Between March and August 2018, 234 participants were included in the validation cohort. Nine of these subjects underwent tissue sampling, eight of whom were diagnosed with lung cancer (adenocarcinoma in situ, *n* = 3; invasive adenocarcinoma, n = 4; small cell carcinoma, *n* = 1); the remaining subjects had benign lesions (pulmonary fibrosis, n = 1). Despite the adoption of identical inclusion criteria, there were several significant differences in demographic features between the training and validation cohorts. The full demographic and clinical descriptions of each cohort at the baseline are presented in Table 1.

For the derivation cohort (*n* = 602), the distribution of baseline Lung-RADS categories was as follows: category 1 (53.66%), category 2 (37.87%), category 3 (5.98%), and category 4 (2.49%). Among the subjects in this cohort, the 19 lung cancer participants (3.16%) included 6 with category 2, 5 with category 3, and 8 with category 4; none had category 1. For the validation cohort (*n* = 234), the distribution of baseline Lung-RADS categories was as follows: category 1 (49.14%), category 2 (46.58%), category 3 (3.00%), and category 4 (1.28%). Among the subjects in this cohort, the 8 lung cancer participants (3.42%) included 7 with category 2 and 1 with category 3; none had category 1 or category 4.

### Performance of prediction models

Using the training set, both the ANN and Lung-RADS showed good discriminative ability with respect to lung cancer risk stratification in the derivation cohort (AUC 0.90 vs. 0.91, respectively, no significant difference). For the Lung-RADS, a sensitivity of 68.4% (95% confidence interval [CI]: 43.4 to 87.4%) and specificity of 93.5% (95% CI: 91.2 to 95.3%) were calculated at the cut-off point of category 3, which adhered to the original definition of a positive LDCT scan. For the ANN, a sensitivity of 73.7% (95% CI: 48.8 to 90.9%) and specificity of 94.7% (95% CI: 92.5 to 96.4%) were calculated at the optimal cut-off value.

**Fig. 3:**
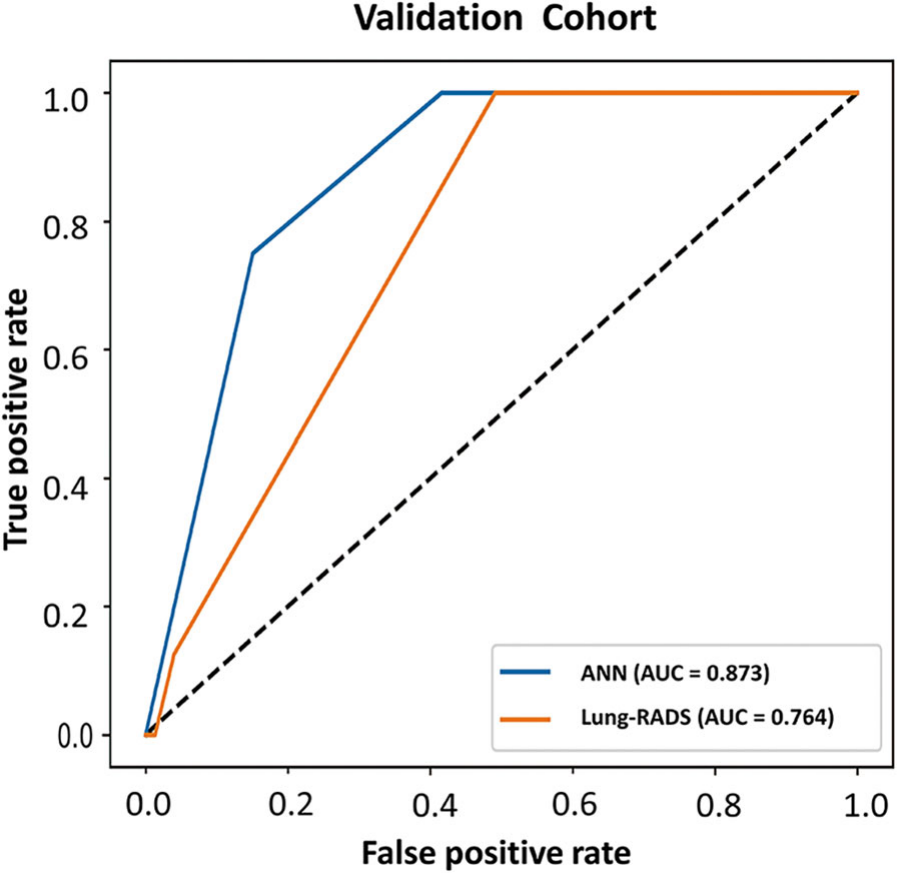
ROC curves for the Lung-RADS and ANN model

Both models were prospectively validated using the validation cohort. Table 2 presents the contingency results of both lung cancer assessment models. Most of the non-cancer cases were correctly identified by both the Lung-RADS and ANN (specificity: 96.0 and 85.0%, respectively), but more lung cancer cases were correctly identified by the ANN (sensitivity: 12.5 and 75.0%, respectively). Figure 3 presents the ROC curves and AUCs for assessing the overall validity of both tools. There was a significant difference between the AUCs of the Lung-RADS and ANN (AUC 0.764 vs. 0.873, respectively, *P* = 0.013). Table 3 presents the sensitivity, specificity, PPV, NPV, LR+, and LR− of the two risk assessment tools. For Lung-RADS, a positive predictive value of 10.0% (95% CI: 1.6 to 43.7%) and negative predictive value of 96.9% (95% CI: 96.0 to 97.6%) were calculated at the cut-off point of category 3. For the ANN, a positive predictive value of 15.0% (95% CI: 9.6 to 22.6%) and negative predictive value of 99.0% (95% CI: 96.7 to 99.7%) were calculated at the optimal cut-off value. The likelihood ratios confirm that the results according to both lung cancer risk classification tools differ from those according to chance.

**Table 2:** Contingency table for the Lung-RADS and ANN models (n = 234)

Scale/model	Lung-RADS			ANN		
	No	Yes	Sum	No	Yes	Sum
Control ^a^	217	9	226	192	34	226
Lung cancer	7	1	8	2	6	8
Sum	224	10	234	194	40	234

^a^ Participant who did not have confirmed lung cancer prior to the index date were labelled as control

**Table 3:** Performance analysis for the Lung-RADS and ANN models (n = 234)

Scale/model	Lung-RADS	ANN
Cut-off	Category 3	> 0.012
AUC (95% CI)	0.764 (0.705, 0.817)	0.873 (0.823, 0.913)
Classification accuracy (%)	93.16	84.62
Sensitivity (95% CI)	12.50 (0.3, 52.7)	75.00 (34.9, 96.8)
Specificity (95% CI)	96.02 (92.6, 98.2)	84.96 (79.6, 89.4)
PPV (95% CI)	10.0 (1.6, 43.7)	15.0 (9.6, 22.6)
NPV (95% CI)	96.9 (96.0, 97.6)	99.0 (96.7, 99.7)
LR+ (95% CI)	3.14 (0.5, 21.9)	4.99 (3.0, 8.3)
LR- (95% CI)	0.91 (0.7, 1.2)	0.29 (0.1, 1.0)

*AUC* area under the curve; *CI* confidence interval; *LR+* positive likelihood ratio; *LR−* negative likelihood ratio; *NPV* negative predictive value; *PPV* positive predictive value

### Feature importance and risk group identification

**Fig. 4:**
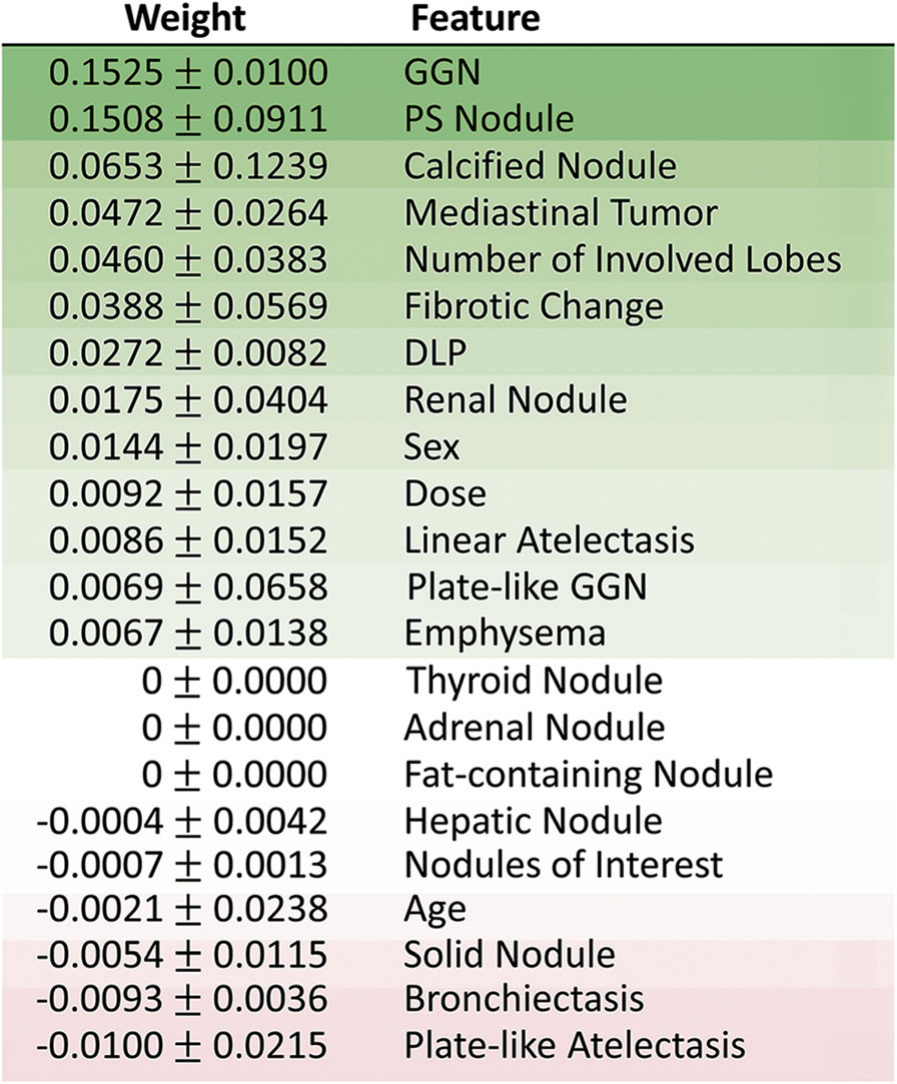
The plot visualizing permutation feature importance scores of the ANN model

Figure 4 shows a plot visualizing permutation feature importance scores of the ANN. In this plot, the rows correspond to the 22 input items of the ANN. The permutation feature importance scores for each feature used in the ANN are calculated and ranked. The items towards the top are the most important features and those towards the bottom matter least. Accordingly, ground-glass nodules (GGNs) and partially solid nodules were important predictors of lung cancer.

## Discussion

In lung cancer screening, LDCT is used to detect pulmonary nodules and evaluate their size and morphology. Most pulmonary nodules are small (< 5 mm in diameter) and benign, and their morphology is variable [24]. Across the lung cancer screening literature, the major challenge faced by this diagnostic imaging modality is the difficulty of defining a “positive scan [25, 26].” The false-positive rate of the Lung-RADS has increased due to the large degree of variation in lung cancer demographics between populations, thus limiting the reliability of this tool [27]. In addition, application of the unitary criteria without appropriate validation may result in false-positive results, overdiagnosis, and unnecessary costs [28]. In this study, the Lung-RADS predicted lung cancer risks for the validation cohort with an AUC of 0.76, which indicated suboptimal decisive power to assess lung cancer risks in the population. The principles of the Lung-RADS are uniformity of radiology interpretation, risk assessment, and nodule management in LDCT lung cancer screening programmes, and although the clinical presentations of lung cancer are likely to vary greatly between populations, some of these imaging findings are not assessed. One possible remedy for this obstacle is the development of a validated prediction model for lung cancer risk using artificial intelligence algorithms, such as ANNs.

Andoni et al. demonstrated the ability of a two-layer neural network to use low-order polynomials [29]. Several studies have used various models to assess the risk of various types of cancer. The results showed that ANN generally achieved better performance than other algorithms [30, 31]. As a preliminary step, we tried to fit the training dataset to several types of models, including ANN, support vector machines, decision trees, naive Bayes classifiers, and linear discriminant classifiers. The ANN showed the best performance, which was comparable to that of Lung-RADS. Therefore, an ANN was used in this study.

In this study, the ANN took many risk factors into account, and it predicted lung cancer risks for the validation cohort with an AUC of 0.87. Compared to the Lung-RADS, ANNs may be more robust in the prediction of lung cancer. Additionally, the standardized structured reports in this study involved the use of lung nodule descriptions from the Lung-RADS lexicon suggested by the ACR. As these input features can be easily identified and are generally assessed by radiologists, the ANN-based LDCT reporting system is both cost-effective and user-friendly.

We also determined predictors of lung cancer; these factors could be useful for identifying patients at high risk of lung cancer. Although previous studies have shown that well-trained ANNs are capable of making accurate predictions for various types of cancer, they have been considered as “black boxes” due to their complexity [30–32]. In this study, efforts were made to determine what the ANN had learnt using the permutation importance estimation method. According to the ranking of permutation feature importance, GGNs and partially solid nodules were important predictors of lung cancer. This study also sought to address the heterogeneity of lung cancer risk assessments in populations containing a high percentage of non-smoking-related lung cancers. Among the subjects in this study, more than one-third of the confirmed lung cancer lesions presented with GGNs < 20 mm (5 of 19 lung cancer cases in the derivation cohort and 5 of 8 lung cancer cases in the validation cohort). When the Lung-RADS was applied, these patients were classified as category 2 and may have been falsely reassured by the “negative” screening results and thus did not return for follow-up scans. Among the 5 of 8 lung cancer cases in the validation cohort, the ANN could identify all (100%) of these patients who had pulmonary lesions and initially presented with GGNs < 20 mm, which were finally confirmed as adenocarcinoma. In several studies performed in Asian cohorts, the majority of lung cancer patients were non-smokers with pulmonary adenocarcinoma spectrum lesions, which typically presented as pure GGNs or partially solid nodules [33, 34]. The current literature shows that larger GGNs (variable cut-off, range 10.5 ~ 15.0 mm) tend to be more aggressive or appear as invasive pulmonary adenocarcinoma [35, 36]. This is a particular concern in Asian populations, where it would be important to report these GGNs and develop corresponding algorithms with follow-up strategies. Therefore, the ANN potentially assimilates population-specific demographic characteristics and provides important insights that improve the efficacy of lung cancer screening programmes.

There were several limitations to this study. First, classification models based on machine learning tend to be unstable in small datasets. However, both models in this study were externally validated using a prospective cohort. Second, the PPVs and NPVs were influenced by the prevalence of disease within the study population. The prevalence of lung cancer was estimated to be approximately 3%, as mentioned above, and is therefore arbitrary to some extent. Finally, the follow-up period was relatively short. A large-scale prospective study with long-term follow-up is required to confirm the benefits of using an ANNs as an element of LDCT lung cancer screening programme.

## Conclusions

Compared to the Lung-RADS, the ANN may have substantially improved the sensitivity for the detection of lung cancer in an Asian population. Furthermore, ANNs have a more refined discriminative ability than the Lung-RADS for lung cancer risk stratification with population-specific demographic characteristics. When lung nodules are detected and documented in a standardized structured report, ANNs may better provide important insights for lung cancer prediction than conventional rule-based criteria. The effects of using an ANN in clinical practice must be examined carefully in further prospective large cohort studies.

## Data Availability

The datasets used and/or analyzed during the current study are available from the corresponding author on reasonable request.

## Abbreviations

ACR: American College of Radiology
ANN: Artificial neural network
AUC: Area under the curve
GGN: Ground-glass nodule
CXR: Chest X-ray
LDCT: Low-dose computed tomography
Lung-RADS: Lung CT Screening Reporting and Data System
NLST: National Lung Screening Trial
NPV: Negative predictive value
PPV: Positive predictive value
ROC: Receiver operating characteristic
